# Co-occurrence of familial cerebral cavernous malformations and tuberculous meningitis

**DOI:** 10.1093/omcr/omaf071

**Published:** 2025-06-27

**Authors:** Bonifacio C Pedregosa II, Herminigildo H Gan, Cyrus G Escabillas, Jose C Navarro

**Affiliations:** Department of Neurology, Jose R. Reyes Memorial Medical Center, Manila City, Philippines; Department of Neurology, Jose R. Reyes Memorial Medical Center, Manila City, Philippines; Department of Neurology, Jose R. Reyes Memorial Medical Center, Manila City, Philippines; Department of Internal Medicine, Far Eastern University - Dr. Nicanor Reyes Medical Foundation, Quezon City, Philippines; Department of Neurology, Jose R. Reyes Memorial Medical Center, Manila City, Philippines; Department of Neuroscience and Behavioral Medicine, University of Santo Tomas Hospital, Manila City, Philippines

**Keywords:** familial cerebral cavernous malformations, Cavernoma, tuberculous meningitis, neurology

A 19-year-old Filipino female presented with generalized tonic–clonic seizures, preceded by a five-week history of progressively worsening holocranial headache accompanied by fever. She was previously well with no known co-morbidities. Neurological examination revealed nuchal rigidity with no other focal deficits. Her father and paternal aunt both had hemorrhagic strokes secondary to cerebral cavernous malformations (CCM).

Biochemical laboratory tests, inflammatory markers, and blood counts were normal. Magnetic resonance imaging demonstrated multiple diffuse CCMs (Fig. 1). Basilar meningeal enhancement and hydrocephalus were also seen. Genetic testing revealed KRIT1 gene mutation, confirming familial CCM. *Mycobacterium tuberculosis* was recovered from CSF cultures, confirming tuberculous meningitis (TBM). To our knowledge, this is the first report describing familial CCM diagnosed concurrently with TBM.

**Figure 1:**
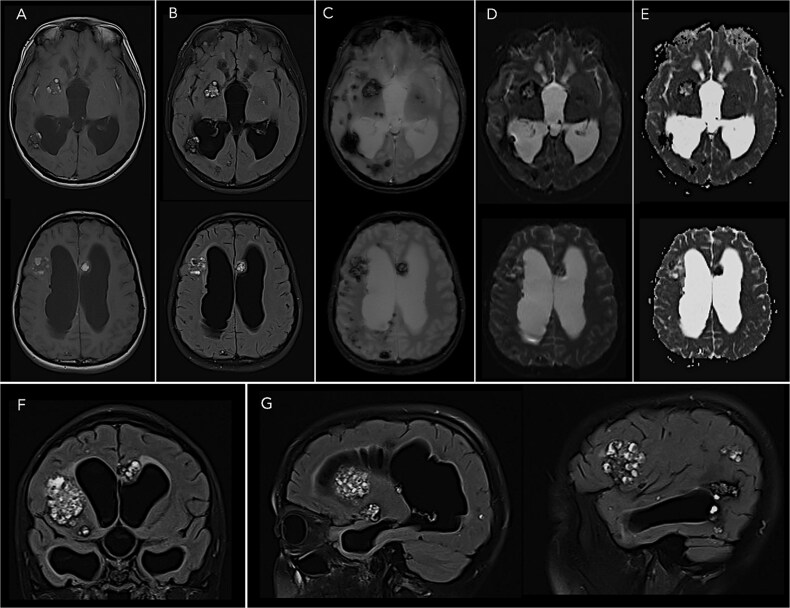
Axial T1*-weighted (A), T2*-weighted fluid-attenuated inversion recovery (B), gradient-recalled echo (C), diffusion-weighted (D), and apparent diffusion coefficient (E) magnetic resonance images demonstrating multiple lobulated complex masses containing blood products of various ages with associated hemosiderin rims consistent with cerebral cavernous malformations in the bilateral cerebral hemispheres, right thalamus, and bilateral capsuloganglionic regions. There is marked dilatation of the ventricular system. Coronal (F) and sagittal (G) T2*-weighted fluid-attenuated inversion recovery magnetic resonance images are also shown.

Characterized by abnormally enlarged capillary cavities without intervening brain parenchyma, CCMs are found in 0.1% to 0.8% of the population [1]. CCMs may occur sporadically as solitary lesions or may be inherited dominantly with incomplete penetrance, usually presenting as multifocal lesions [2]. Familial CCMs constitute 20% of cases and are associated with somatic mutations in one of three CCM genes, with KRIT1 mutations accounting for majority of these cases [2]. CCMs are managed either conservatively or surgically when accessible lesions cause recurrent hemorrhage or seizures [2].

TBM develops when focal tuberculous lesions in communication with the meninges rupture, triggering a dysregulated inflammatory response [3]. In developing countries, TBM is one of the most common causes of subacute to chronic meningitis [3]. Early initiation of antitubercular treatment and corticosteroids increases the likelihood of favorable outcomes; CSF diversion should be considered for progressive hydrocephalus [3, 4].

The patient underwent ventricular drainage and was started on antitubercular agents, corticosteroids, and anti-seizure medications. Her neurological deficits eventually resolved without seizure recurrence. Surveillance neuroimaging revealed regression of hydrocephalus, without any interval change in the size of the CCMs.

## Consent

Written informed consent was obtained from the patient for publication of this report and any accompanying images.

## Guarantor

Bonifacio C. Pedregosa II, MD.
